# *In vivo* production of CAR T cell: Opportunities and challenges

**DOI:** 10.1016/j.gendis.2025.101612

**Published:** 2025-03-25

**Authors:** Zhiqiang Song, Yi Zhou, Binbin Wang, Yuke Geng, Gusheng Tang, Yang Wang, Jianmin Yang

**Affiliations:** a Department of Hematology, Institute of Hematology, Shanghai Changhai Hospital, Naval Medical University, Shanghai 200433, China; b Department of Hematology, Myeloma & Lymphoma Center, Shanghai Changzheng Hospital, Naval Medical University, Shanghai 200433, China; c Department of Oncology, No. 970th Hospital of Joint Logistic Support Force of PLA, Yantai, Shandong 264000, China

**Keywords:** Chimeric antigen receptor T cell, Hematological malignancies, *In vivo* production, Nonviral vector, Viral vector

## Abstract

Chimeric antigen receptor T (CAR T) cell therapy has achieved remarkable efficacy for patients with hematological malignancies. However, *in vitro* viral vector-mediated production of CAR T cells is time-consuming and expensive and impairs T cell function. On one hand, an elaborate manufacturing process not only impairs the function of CAR T cells but also limits its usage in patients with rapidly progressing diseases. On the other hand, high costs are incompatible with broad clinical applications for sizable populations. *In vivo* production of CAR T cells is a novel approach that can avoid complicated production processes and reduce costs through mass production. Additionally, *in vivo* production of CAR T cells does not damage the function of T cells compared with *in vitro* production. Early studies have demonstrated promising antitumor activity of *in vivo* CAR T cell therapy in preclinical models of hematological malignancies. In this review, we describe the latest developments of *in vivo* CAR T cell therapy and discuss its potential challenges for clinical application.

## Introduction

Chimeric antigen receptor T (CAR T) cell therapy has achieved remarkable clinical efficacy in patients with relapsed/refractory hematological malignancies. In pivotal clinical trials, the overall response rates were 81% and 71% in patients with relapsed/refractory B-cell acute lymphoblastic leukemia following tisagenlecleucel and brexucabtagene autoleucel therapies,^1^^,^^2^ respectively. Similarly, CAR T cell therapy also presented impressive overall response rates for patients with relapsed/refractory large B-cell lymphomas, ranging from 53% to 83%.^3^, ^4^, ^5^ These results were significantly superior to those of traditional chemotherapy, with a poor complete response rate (7%) and 2-year overall survival (20%).^6^ CAR T cell therapy has also achieved promising clinical efficacy in patients with relapsed/refractory multiple myeloma.^7^^,^^8^ The indications and treatment responses of different U.S. FDA-approved CAR T cell therapies are presented in Table 1. These promising results contributed to the U.S. FDA's approval of four CD19 CAR T cell therapies: tisagenlecleucel, axicabtagene-ciloleucel, brexucabtagene autoleucel, and lisocabtagene maraleucel; however, these four CAR T cell therapies are expensive ($475,000, $373,000, $373,000, and $410,300, respectively).^9^, ^10^, ^11^ Moreover, these costs do not include hospitalization fees. Consequently, many patients fail to benefit from CAR T cell therapy due to the unaffordable costs.

**Table 1:** The indications and treatment responses of different CAR T cell therapies for hematological malignancies.

CAR T cell therapy	Target	Indication	ORR (%)	CR (%)	Median OS (M)	Reference
Tisagenlecleucel	CD19	R/R B-ALL (≤25)	81	60	19.1	Maude et al^1^
		R/R LBCL	53	39	11.1	Schuster et al^3^
		R/R FL	86.2	69.1	NR	Fowler et al^25^
Axicabtagene-ciloleucel	CD19	R/R LBCL	83	58	NR	Locke et al^136^
		R/R FL	94.2	79.1	NR	Jacobson et al^137^
Lisocabtagene maraleucel	CD19	R/R LBCL	73	53	21.1	Abramson et al^138^
Brexucabtagene autoleucel	CD19	R/R B-ALL	69	53	12.1	Shah et al^139^
		R/R MCL	91	68	46.6	Wang et al^140^
Idecabtagene vicleucel	BCMA	R/R MM	73	33	19.4	Munshi et al^7^
Ciltacabtagene autoleucel	BCMA	R/R MM	97	67	NR	Berdeja et al^8^

Note: CAR, chimeric antigen receptor; ORR, overall response rate; CR, complete response; OS, overall survival; R/R, relapsed or refractory; NR, not reached; ALL, acute lymphoblastic leukemia; LBCL, large B-cell lymphoma; MCL, mantle cell lymphoma; MM, multiple myeloma.

**Figure 1:**
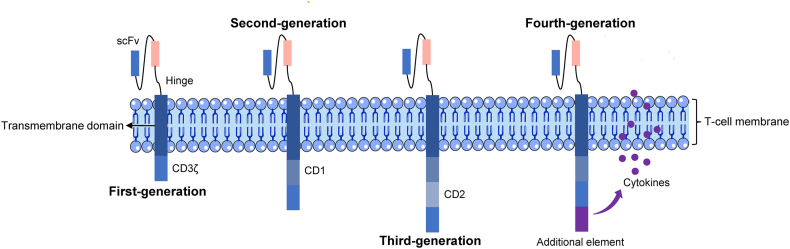
The basic structure of five generations of CAR T cells. The first-generation CAR T only includes CD3ζ as an intracellular signal and the second-generation CAR T adds a co-stimulatory domain (CD1) based on the first-generation. The third-generation CAR T contains two co-stimulatory domains (CD1 and CD2); the fourth-generation CAR T includes a controllable on-off switch or additional element to enhance the function by secreting the cytokines; the fifth-generation CAR T is universal by combining the gene editing technologies such as CRISPR/Cas9. CAR T, chimeric antigen receptor T; scFv, single chain variable fragment; CD, co-stimulatory domain.

Chimeric antigen receptor (CAR) is a genetically modified receptor that mainly consists of four components: an extracellular domain, a hinge region, a transmembrane domain, and an intracellular signaling domain (Fig. 1).^12^ The extracellular domain contains a single chain variable fragment (scFv) to specifically bind to antigens independent of human leukocyte antigen, and the hinge region can affect CAR expression and epitope recognition.^13^^,^^14^ The transmembrane domain anchors the CAR to the T cell membrane and affects the stability and expression levels of CAR.^15^^,^^16^ The intracellular signaling domain includes one or two co-stimulatory domains linked to CD3ζ driving immunoreceptor tyrosine-based activation motifs.^17^ Currently, the most common co-stimulatory domains are CD28 and 4-1BB, and they have achieved unprecedented success in treating hematological malignancies.^18^^,^^19^ Additionally, other co-stimulatory signaling molecules have also demonstrated potent anti-tumor activity in preclinical research, such as OX40, inducible costimulatory (ICOS), and CD27.^20^, ^21^, ^22^

Notably, the lack of co-stimulatory domains is unable to activate T cells, which resulted in insufficient efficacy of the first-generation CAR T cell.^23^^,^^24^ In contrast, the second-generation CAR T cell includes a co-stimulatory domain (CD), and its anti-tumor ability is significantly improved.^25^^,^^26^ The third-generation CAR T cell contains two CDs and its anti-tumor activity should theoretically be stronger compared with the second-generation CAR T cell. However, the results of published studies were arguable. CAR T cell with two CDs (CD28 and 4-1BB) exhibited superior proliferation capacity and anti-tumor function in treating multiple myeloma and relapsed/refractory non-Hodgkin's lymphoma compared with CAR T cell with CD28,^27^^,^^28^ while the third-generation of CAR T cell did not present stronger anti-tumor ability in prostate and pancreatic cancers.^29^^,^^30^ The mechanisms underlying these conflicting results warrant further investigation. Of note, the fourth-generation CAR T cell is armed to secrete various cytokines to overcome tumor microenvironment,^31^^,^^32^ providing new hope for killing solid tumors.

Generally, CAR T cell is produced through the following four steps^33^: i) isolation and collection of T cell from patients by leukapheresis; ii) activation and genetic modification of T cell with CAR structure; iii) expansion of modified T cell to achieve approximately 10^7^–10^9^ cells and cryopreservation; and iv) quality control examination of CAR T cell, lymphodepletion chemotherapy, and infusion of CAR T cell. This laborious manufacturing process takes 2–3 weeks and can be summarized as “one patient, one batch”, which limits scalable production and application for patients with rapidly progressive diseases.^3^^,^^10^ In this review, we aimed to highlight the recent advancements of *in vivo* CAR T cell therapy (summarized in Table 2), and discussed its potential challenges for clinical application.

**Table 2:** Summary of *in vivo* production of CAR T cells.

Delivery vector	Vector	Cargo	Target	Disease	Result	Reference	Limitation
Viral vector	LV	CD19 CAR plasmid	CD8	Raji lymphoma	Effective elimination of CD19^+^ B cells and cytokine release syndrome	Pfeiffer et al^82^	Immunogenicity and insertional oncogenesis
	LV	CD19 CAR plasmid	CD8	Nalm-6 leukemia	Eliminating the tumor cells from bone marrow and spleen and CAR NK cells were also observed	Agarwal et al^49^	
	LV	CD19 CAR plasmid	CD4	Nalm-6 leukemia	Superior anti-tumor activity than CD8-targeted LV	Agarwal et al^83^	
	LV	CD19 CAR plasmid	CD3	–	Efficient and exclusive transduction of CD3^+^ T cells	Frank et al^84^	
	LV	CD19 CAR plasmid	Mutant E2 glycoprotein and CD3	Aggressive BV-173 B cell lymphoma	Markedly reducing the growth of B cell tumor	Huckaby et al^86^	
	LV	CD19 CAR plasmid	Murine CD8	A20 B cell lymphoma	Elimination of B lymphocytes and lymphoma cells	Michels et al^87^	
	AAV	Hu5A8 CAR plasmid	CD4	MT2 adult T-cell leukemia	Tumor regression	Nawaz et al^79^	
Nanoparticle vector	Polymer/lipid-based reagent	CD3 × CLDN6 mRNA	–	OV-90 ovarian carcinoma	Eliminating OV-90 cancer cells upon consecutive injection	Stadler et al^119^	Low delivery efficiency and strict production rules
	LNP	Against FAP CAR mRNA	CD5	Heart failure	Reducing fibrosis and restoring cardiac function after injury	Rurik et al^51^	
	PBAE	ROR1 and CD19 CAR mRNA	CD8 or CD3	Eμ-ALL01 leukemia and LNCaP C42 prostate adenocarcinoma	Anti-tumor ability was similar to *in vitro* CAR T therapy	Parayath et al^52^	
	PBAE	CD19 CAR plasmid	CD3	Eμ-ALL01 leukemia	Anti-leukemia activity was similar to *in vitro* CAR T therapy	Smith et al^41^	
	PAMAM and PEI	EGFRvIII CAR plasmid	–	HuH7 hepatocarcinoma	Recognizing and binding specifically with EGFRvIII-positive tumor cells	Yu et al^124^	

Note: LV, lentiviral vector; AAV, adeno-associated virus; CAR, chimeric antigen receptor; CAR T, chimeric antigen receptor T; FAP, fibroblast activation protein; NK, natural killer; PAMAM, polyamidoamine; PEI, polyethyleneimine; LNP, lipid nanoparticles; PBAE, Poly(β-amino ester); EGFRvIII, epidermal growth factor receptor variant III. “—" means “not applicable".

## Increasing clinical needs of *in vivo* CAR T cell therapy

### “Off-the-shelf” products

The elaborate manufacturing process of *in vitro* CAR T cells is one of the major roadblocks that limit its broad clinical application. On the one hand, this “one patient, one batch” personalized treatment undoubtedly increases the cost of CAR T cell therapy; on the other hand, a long waiting time between T cell isolation and CAR T cell infusion decreases the efficacy of CAR T cell therapy.^34^ Previous studies have explored shortening CAR T cell preparation time from 2–3 weeks to 1 day by improving transduction efficiency and implanting simulated biological scaffolds.^35^^,^^36^ However, a 1-week quality control analysis is still necessary for these optimized CAR T cell therapies before infusion to patients. Compared with autologous CAR T cell therapy, universal CAR T cells from healthy donors can be made in bulk quantities and are available for patients quickly. With robust genome editing technologies, such as clustered regularly interspaced short palindromic repeats (CRISPR) and CRISPR-associated protein 9 (Cas9), universal CAR T cell therapy has resulted in remarkable success in previous studies.^2^^,^^37^^,^^38^ Nevertheless, this treatment strategy not only alters the phenotypes and activities of T cells but also unavoidably increases the costs, including the intellectual property of genome editing technologies.

By contrast, *in vivo* production of CAR T cell creates “off-the-shelf” products, which can be made available at any time, like various antibody drugs such as blinatumomab (a CD3/CD19-directed bispecific T-cell engager).^39^ However, blinatumomab needs to be administered repeatedly, which can lead to fatal adverse events, such as sepsis, *Escherichia coli* sepsis, and Candida infection.^40^ Moreover, blinatumomab cannot achieve active biodistribution or expand to function continuously after injection into the body. Conversely, *in vivo* CAR T cell therapy can target diseases actively, self-amplify, and kill tumor cells constantly.^41^ Therefore, *in vivo* CAR T cell therapy has the advantages of both CAR T cell therapy and antibody drugs, which show rapid and constant anti-cancer efficacy and are suitable for patients with rapidly progressive diseases.

### Feasibility

Unlike *in vitro* CAR T cell production with a strenuous process and high costs, large-scale production of *in vivo* CAR T cells is feasible due to the availability of good manufacturing practice (GMP) platforms for nanoparticles, which are important CAR delivery vectors *in vivo*.^42^^,^^43^ These platforms can produce nanoparticles up to gram amounts in 1 day and they can be stored in a stable form using lyophilized reagents, which largely reduce the costs of the nanoparticles. Therefore, nanomedicines can be made at an inexpensive price, making it possible for a sizable population to receive CAR T cell therapy. Of note, published studies have proved that nanoparticles carrying nucleic acid are stable for *in vivo* genome editing,^44^^,^^45^ while *in vitro* production of CAR T cells is highly diverse and variable due to the “one patient, one batch” treatment strategy and complicated manufacturing process.^3^^,^^46^^,^^47^ Furthermore, lymphodepletion chemotherapy is not mandatory in *in vivo* CAR T cell therapy because reprogrammed T cell is consistently located in the physiological environment and is never stimulated by supraphysiological cytokines.^3^ In addition, lymphodepletion chemotherapy makes patients susceptible to severe infectious complications due to lymphodepletion-associated hematotoxicity.^46^^,^^48^ Therefore, lymphodepletion chemotherapy-associated toxicities could theoretically be avoided in the *in vivo* CAR T cell therapy.

**Figure 2:**
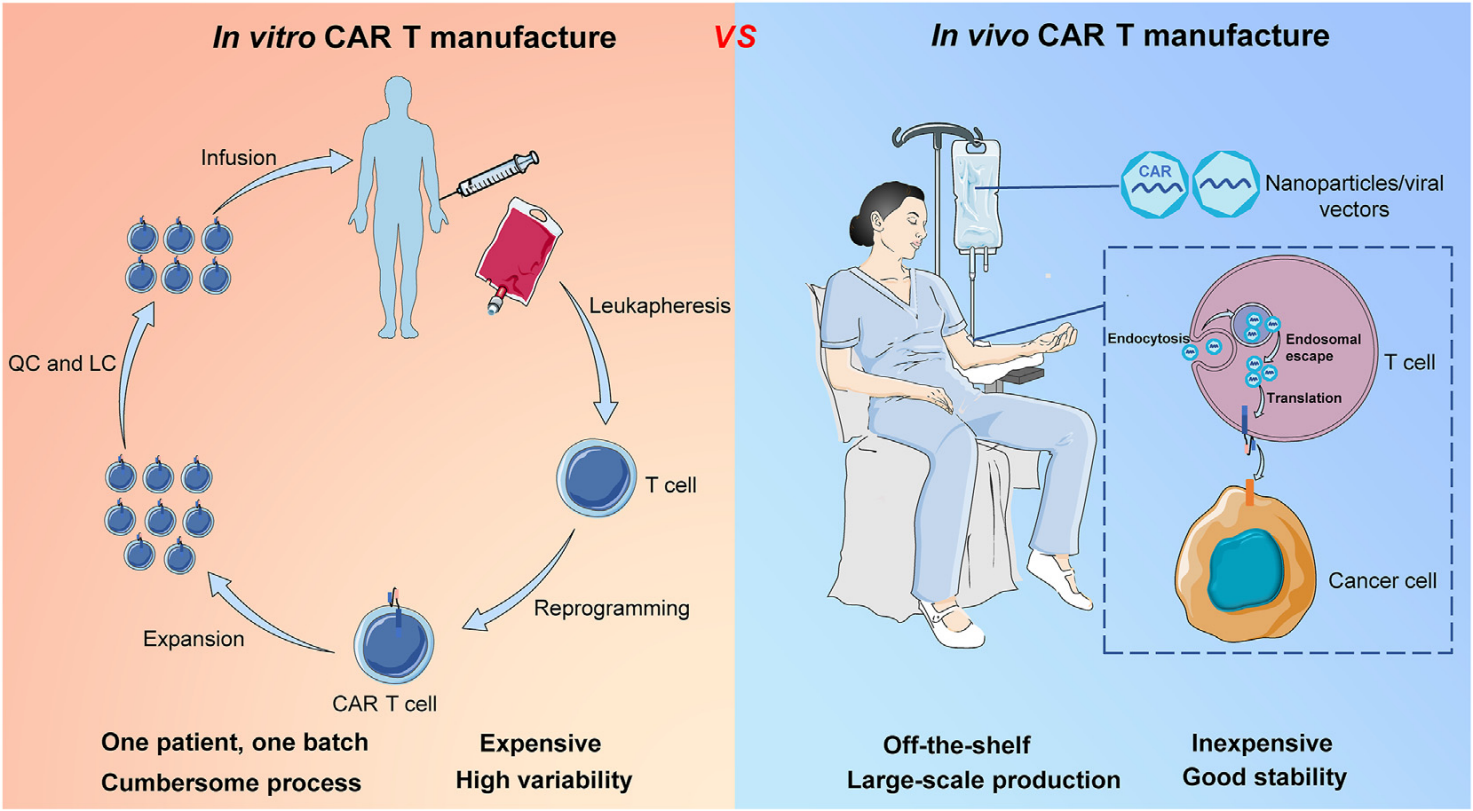
The comparison of *in vitro* and *in vivo* CAR T cell therapy. Generally, *in vitro* production of CAR T cells is comprised of the following four steps: isolating T cells by leukapheresis, reprogramming, expansion, and quality control (QC) analysis. Additionally, lymphodepletion chemotherapy (LC) is necessary before CAR T cell infusion. *In vitro* production is “one patient, one patch”, expensive, cumbersome, and highly variable. Correspondingly, *in vivo* production of CAR T cells only needs to infuse the nanoparticles or virus-carrying CAR, which is “off-the-shelf”, inexpensive, and stable, and can be produced on a large scale. CAR T, chimeric antigen receptor T.

Currently, various vectors have been successfully applied to produce *in vivo* CAR T cells and they are mainly divided into two categories, viral vector and nonviral vector. The former includes lentiviral vector (LV)^49^ and adeno-associated virus (AAV),^50^ while the latter mainly includes nanoparticles.^51^ In addition, there are multiple cargoes for *in vivo* CAR T cell production, ranging from the transient expression of messenger RNA (mRNA)^52^ and small interfering RNA (siRNA)^53^ to persistent modified gene plasmid DNA.^41^ In short, *in vivo* CAR T cell therapy is a feasible and promising strategy to overcome the limitations of *in vitro* CAR T cell therapy (Fig. 2).

### *In vivo* production techniques

A variety of techniques have been successfully applied to genome editing *in vivo*, and these are composed of *in vivo* viral and nonviral production techniques, including AAV, LV, electroporation, nanoparticles, and genetically engineered proteins. The major advantages and challenges of various *in vivo* production techniques are summarized in Table S1.

### *In vivo* viral production techniques

Over the past decade, multiple viral vectors have been used to perform reprogramming, including retroviral vector, LV, adenoviral vector, AAV, Sendai viral vector, and baculoviral vector.^54^, ^55^, ^56^, ^57^ However, LV and AAV are the most frequently used vectors owing to their high transfection rates and low safety concerns. As a single-stranded non-pathogenic DNA parvovirus, the AAV genome consists of Rep and Cap genes, which are essential for genome replication and capsid assembly, respectively.^58^ AAV includes a capsid carrying up to ∼4.4 kb of external genome flanked by cis-elements, and they have been successfully utilized for gene reprogramming to a variety of tissues *in vivo*.^59^ Zhao et al proved that *in vivo* gene editing of Ldlr^E208X^ after administration of a single AAV-CRISPR/Cas9 could ameliorate atherosclerosis phenotypes of familial hypercholesterolemia.^60^ Tabebordbar et al found that the restoration of dystrophin was achieved by a one-time injection of AAV-Duchenne muscular dystrophy CRISPR, and partially recovered muscle function in a mouse model.^61^ In addition, AAV has been used for treating central nervous system diseases^62^ and rescue auditory function.^63^ The dose of AAV used for transduction is not pathogenic, and a low integration rate decreases the risk of insertional mutations,^64^ However, the limitations of AAV are limited packaging size and vector immunogenicity.^65^^,^^66^

Lentivirus, a subtype of the retrovirus family, is a single-stranded RNA virus that can use reverse transcriptase and integrase to stably insert genome into dividing and non-dividing cells. Lentivirus is a replication-incompetent vector mostly originating from HIV-1 without the ability to express viral proteins, and they can typically carry ∼10 kb of exogenous genome information.^67^^,^^68^ To date, LV has been harnessed for *in vivo* gene editing to treat a variety of diseases. Ling et al utilized LV co-delivery of *Streptococcus pyogenes* Cas9 mRNA and guided RNA targeting vascular endothelial growth factor A (VEGFA) and efficiently knocked out 44% of VEGFA, which provided a new therapy for retinal neovascular diseases.^69^ Similarly, LV combined with CRISPR/Cas9 techniques has also been successfully applied to treat Alzheimer's disease.^70^ Although the carrying capacity of LV is significantly larger than that of AAV, the disadvantages of LV are fewer cellular targets and insertional mutations compared with AAV.^71^

### *In vivo* nonviral production techniques

A range of nonviral techniques have been developed to perform gene editing *in vivo*. Suzuki et al demonstrated the feasibility of *in vivo* electroporation of muscles and kidneys via CRISPR/Cas9 technology to achieve target gene integration.^72^ However, this technique is difficult to perform in clinical studies due to unclear electroporation parameters and unavoidable tissue damage. A variety of nanoparticle strategies have been used to perform genome editing *in vivo*, including solid lipid nanoparticles, cationic lipids, and gold nanoparticles. Yin et al showed that a single administration of solid lipid nanoparticles carrying chemically modified single guide RNA (sgRNA) and Cas9 mRNA could induce more than 80% editing of proprotein convertase subtilisin/kexin type 9 (Pcsk9) in the liver and lower cholesterol levels in mice.^73^ Cationic lipid-mediated *in vivo* delivery can rescue autosomal dominant hearing loss in a mouse model.^74^ Similarly, gold nanoparticles carrying the CRISPR/Cas9 ribonucleoprotein provided a new treatment for rescuing fragile X syndrome.^75^ Although nanoparticles are a promising platform for delivering transgenes *in vivo*, particularly in combination with other techniques such as CRISPR/Cas9, the clinical translation of this technique also needs to overcome some challenges. For example, the toxicity of cationic lipids significantly hinders their further application in clinical trials, and the development of safer biocompatible lipids for gene delivery *in vivo* is imperative.^76^ Additionally, the transfection rates of nanoparticle delivery are not as high as those of viral techniques, and repeat administration is often necessary to cure diseases, especially for transient transfection. Finally, the combined application of CRISPR/Cas9 may cause large deletions and complex rearrangements, leading to pathogenic consequences.^77^

In addition to the above *in vivo* production techniques, engineering proteins is a direct and feasible method for performing genome editing *in vivo*. Staahl et al demonstrated that the direct injection of engineered Cas9 ribonucleoprotein complexes could achieve efficient neuronal editing *in vivo* and provide a new therapy for various neurological diseases.^78^ Such engineered proteins have strong potential for *in vivo* genome editing. For example, the size of these genetically constructed proteins is between 5 and 10 nm, significantly smaller than nanoparticles, which ensures their extravasation from the blood to tissues. In addition, the production of these engineered proteins is much easier than that of nanoparticles owing to the presence of single and well-defined substances. However, preexisting human immunogenicity to external proteins is a major challenge to the clinical efficiency of engineered proteins.^59^

## *In vivo* production of CAR T cell by viral transfection

### Rationale

**Figure 3:**
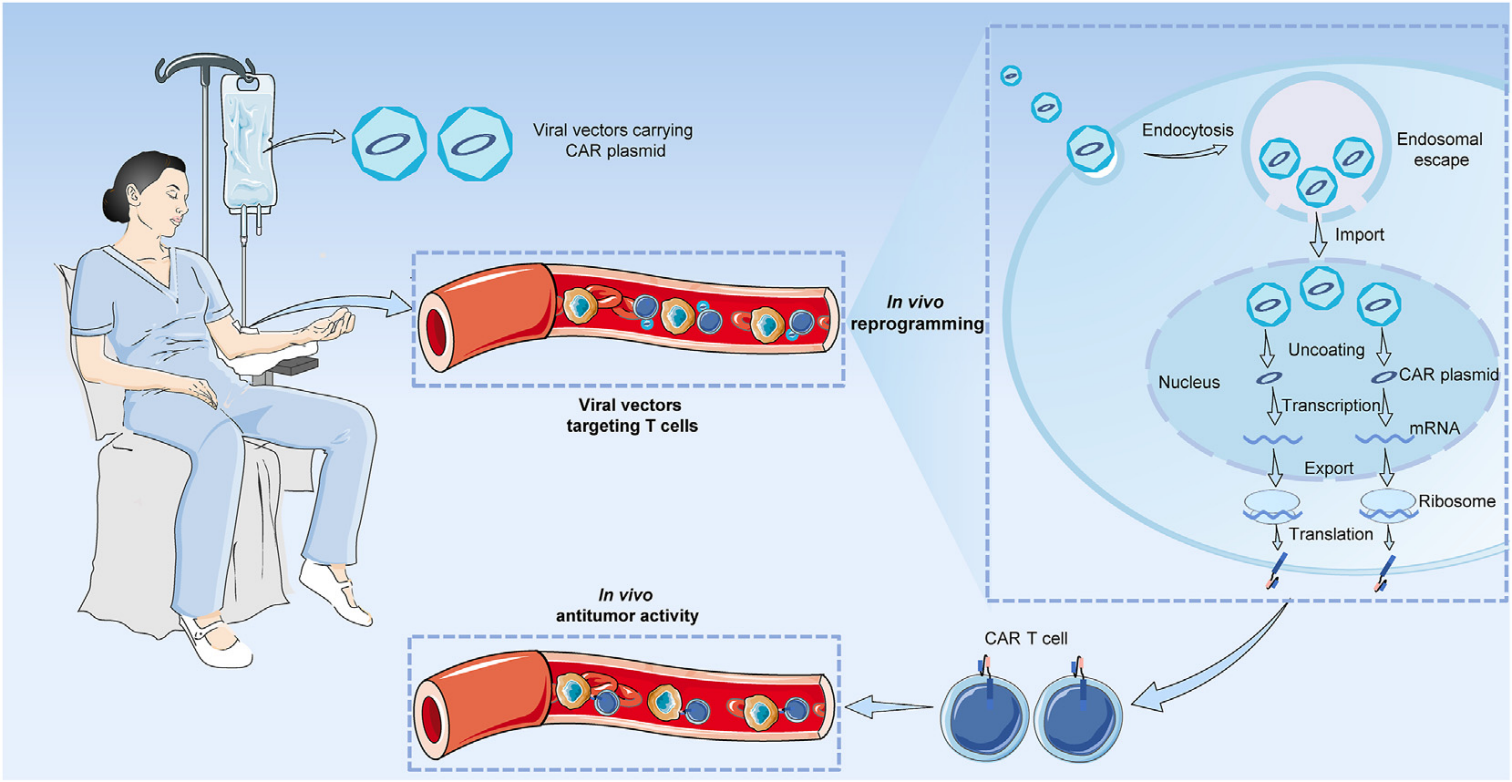
The schematic illustrating the process of *in vivo* production of CAR T cell by viral vector carrying disease-specific CAR plasmid. The viral vector is T cell-targeted, so it can specifically bind to T cells and transfer the CAR plasmid into T cells after entering the body. Then, it enters the nucleus to complete the reprogramming, express the disease-specific CAR on the surface, and kill the tumor cells *in vivo*. CAR, chimeric antigen receptor; CAR T, chimeric antigen receptor T.

At present, LV is the most widely used transgene-bearing vector to produce CAR T cells *in vitro*, owing to their efficient transfection ability and stable characteristics of gene transfer for non-dividing cells.^67^ Additionally, LV has been successfully applied in the production of the first U.S. FDA-approved CD19-targeted CAR T cell therapy tisagenlecleucel,^1^ further suggesting their favorable safety in humans. Similarly, AAV is also frequently used to manipulate CAR and other gene transfections to cure multiple diseases and has been utilized in approximately 200 clinical trials to date.^79^, ^80^, ^81^ Moreover, both LV and AAV could carry CAR genes to produce CAR T cells *in vivo* (Fig. 3), reducing the patients' waiting rounds, and they can be made available immediately for rapidly progressive diseases, thus, preventing disease progression.

### LV transfection

To date, LV-carrying CAR transgene has achieved great success in preclinical studies. Pfeiffer and colleagues first produced human CD19-targeted CAR T cells *in vivo* using CD8-targeted LV selectively delivering CD19 CAR into CD8^+^ T cells, effectively eliminating CD19^+^ B cells in the NOD-scid-IL2Rγnull (NSG) mouse Raji lymphoma model.^82^ To imitate the human physiological environment, they further infused CD8-targeted LV carrying CAR into NSG mice transplanted with human CD34^+^ hematopoietic stem cells, and CAR T cell was detected in 7 out of 10 mice, accompanied by CD19^+^ B-cell aplasia due to increased inflammatory cytokine levels. This research group also produced CAR T cells *in vivo* to eliminate CD19^+^ Nalm-6 tumor cells upon administration of CD8-targeted LV delivering CD19-CAR.^49^ Of note, a small portion of CAR^+^ natural killer cells (NK and NKT) were also observed, which could further enhance the anti-tumor ability of CAR T cells. In addition, the same group investigated the production of CD4^+^ CAR T cells *in vivo* using CD4-targeted LV, which showed a faster and superior tumor-eliminating capability compared with CD8-targeted LV in NSG mice reconstituted with human CD34^+^ cells, mainly because CD8^+^ T cells are more likely to be exhausted.^83^ Buchholz's research team made a step forward by introducing a novel CD3-targeted LV capable of achieving human T cell gene transfection without prior activation *in vivo*.^84^ Although the abilities of activation, proliferation, and expansion of this CD3-targeted LV were not as good as those of conventional *in vitro* activation due to the lack of costimulatory molecules, it could genetically modify non-activated T cells without any additional external stimuli and successfully deliver CD19 CAR into CD3^+^ T cells *in vivo* in humanized NSG mice. In addition, this team also provided detailed protocols for manufacturing CD4-and/or CD8-targeted LV and discussed how the protocol can be easily adapted to produce LV targeting other tumor antigens *in vivo*.^85^

To reduce the transfection of nontarget cells, Huckaby et al redirected the LV to CD3^+^ human T cells using a bispecific antibody binder.^86^ They used a mutated Sindbis pseudotyped LV with mutant E2 glycoprotein lacking its inherent tropism to human cells and incorporated the bispecific binder redirecting mutant E2 glycoprotein and CD3 molecule, and the modified CAR T cell *in vivo* markedly reduced the tumor burden in xenograft B cell tumor models. In a study performed by Michels and coworkers, they designed ankyrin repeat proteins (DARPins) binding to murine CD8 (mCD8) to successfully produce mCD8-targeted LV and AAV exhibiting >99 % specificity for CD8^+^ cells.^87^ Furthermore, Frank et al found that DARPins could improve specific gene transfer efficacy in human and primate T lymphocytes.^88^ These findings suggest that the modified targeting receptor could enhance the specificity of transfection, and thus reduce the “off-the-target” effect. Notably, Nicolai et al utilized the LV to successfully generate anti-CD20 CAR T cells in nonhuman primates and achieve complete B-cell depletion for over 10 weeks.^89^

### AAV transfection

As a small single-stranded DNA virus, AAV consists of rep and cap genes flanked by two inverted terminal repeats.^58^ In recent years, AAV-mediated gene therapy technology has been extensively applied in clinical trials for various diseases, such as neurological,^90^ muscular,^91^ and ocular diseases.^92^ AAV-mediated gene delivery is currently used to construct diverse target-specific CAR T cells *in vitro*. Sather et al introduced AAV-carrying anti-HIV-CAR and anti-CD19-CAR into the CCR5 locus of primary T cells via megaTAL nucleases, resulting in 14% and 9% insertion efficiencies, respectively. It exhibited significant anti-HIV or antitumor responses.^93^ Combined with CRISPR/Cas9 technology, Eyquem et al incorporated the AAV6 vector-delivered anti-CD19-CAR into the TRAC locus of T cells and achieved a CAR insertion rate of more than 40%, showing more potent efficacy in killing CD19^+^ leukemia cells than conventional retrovirally produced CAR T cell.^94^ MacLeod et al produced allogeneic CAR T cells by target-inserting a CAR transgene into the T-cell receptor locus using an AAV donor template and endonuclease, making it possible for patients with advanced disease or insufficient CAR T cells to receive CAR T cell therapy.^95^

Good safety profile and potent efficacy have promoted AAV as a good vector to produce CAR T cells *in vivo*. A recent study produced CD4^+^ CAR T cells *in vivo* by injecting the AAV-carrying CAR gene, resulting in anti-tumor immunological characteristics and potent efficacy against human T-cell leukemia.^79^ Nawaz et al reported their AAV-CD4CAR with Hu5A8 antibody targeting CD4 and demonstrated anti-leukemia activity by infusing AAV-CD4CAR into humanized NOD. Cg-Prkdcscid Il2rgem26/Nju mice bearing T-cell leukemia.^96^ However, this study did not evaluate CAR expression in other immune and non-immune cells, leading to concerns about the specificity of AAV-mediated gene therapy.

### Limitations of viral transfection

Although promising results have been observed in preclinical studies of CAR T cell production *in vivo* via viral transfection, the following challenges are also encountered: safety concerns, high costs, off-target effects, and immunogenicity. Both LV- and AAV vector-mediated gene therapy present high safety levels and transfection efficacy, however, they prefer to integrate into highly transcribed and cancer-related genes, leading to a potential risk of insertional oncogenesis.^97^^,^^98^ This question is particularly critical for *in vivo* CAR T cell therapy, given that viral particles are directly injected into patients. An interesting study about bacteria-free minicircle vectors to produce integration-free CAR T cells was reported by Cheng and coworkers.^99^ Although the time of CAR expression via the minicircle vector is significantly shorter than that of LV and AAV vectors, it can eliminate cancer cells efficiently and avoid insertional oncogenesis. Therefore, the minicircle vector might be a better choice for constructing CAR T cells *in vivo* in terms of safety.

In addition, the high cost and regulatory requirements of LV and AAV are incompatible with their rapid and broad clinical applications for sizable patient populations.^100^ Wang et al explored a large-scale platform for generating cytomegalovirus (CMV)-CD19CAR T cells from CMV-specific T cells and anticipated that CMV-CD19CAR T cells could expand *in vivo* after injection of the CMV-modified Vaccinia Ankara Triplex vaccine.^101^ This large-scale CAR T cell manufacturing platform may largely reduce costs and allow more patients to benefit from gene therapy. The biodistribution and off-target effects in different tissues were not analyzed following LV and AAV vector injection in the abovementioned studies, which is also a major concern for their wide usage in the future. Additionally, immunogenicity limits the extensive application of viral vector-based *in vivo* gene therapy, leading to a decline in transduction efficiency, elimination of transduced cells, and decreased vector stability.^102^ Milani et al incorporated the human phagocytosis inhibitor, CD47, into the LV to reduce the uptake of viral vector by phagocytes and the innate immune system.^103^ Reducing or eliminating immunogenicity is necessary for patients receiving *in vivo* gene therapy, especially for those with advanced diseases, because they might need higher doses.

## *In vivo* production of CAR T cell by nonviral transfection

### Rationale

**Figure 4:**
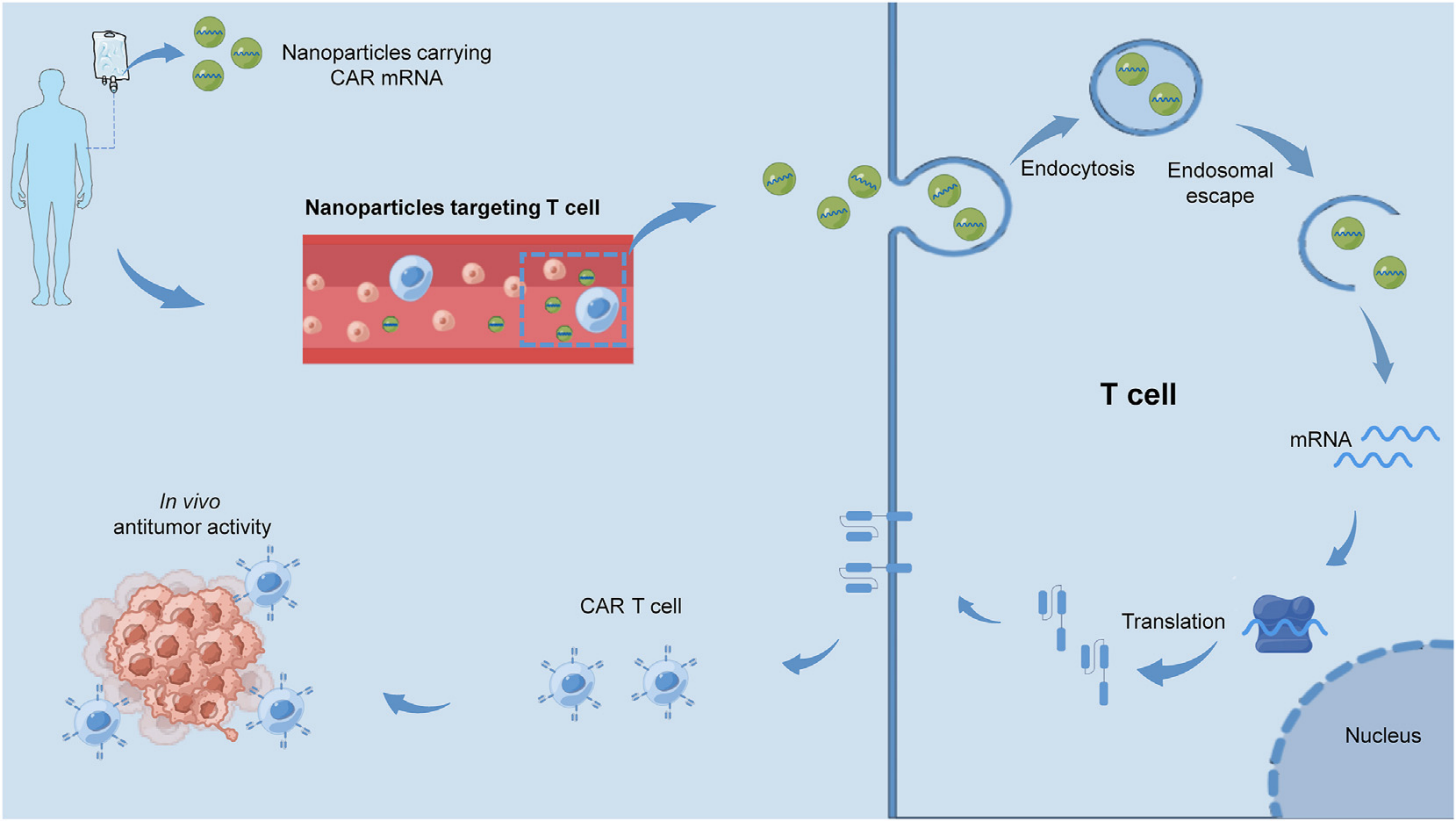
The schematic illustrating the process of *in vivo* production of CAR T cell by nanoparticles carrying disease-specific CAR mRNA. These nanoparticles are T cell-targeted, so they can specifically bind to T cells and transfer the CAR mRNA into T cells after infusing into the body. Then, they complete the reprogramming in the cytoplasm, express the disease-specific CAR on the surface, and kill the tumor cells *in vivo*. CAR, chimeric antigen receptor; CAR T, chimeric antigen receptor T.

In addition to viral transfection, nonviral transfection is also a promising gene editing strategy to produce CAR T cells, such as electroporation, sleeping beauty transposition,^104^ transposon piggyBac,^105^ and nanoparticles.^41^ Among these nonviral transfection technologies, electroporation has been proven to be a feasible and effective tool to directly introduce CAR mRNA into T cells *in vitro*.^106^^,^^107^ However, the electroporation process involves substantial costs and requires extensive infrastructure. Like virus-based gene transfer, transposon systems carry the potential risk of insertional mutations.^108^^,^^109^ In addition, transposons must depend on other gene delivery methods to enter the cells, such as electroporation, which undoubtedly increases the complexity and cost of manufacturing CAR T cells. Compared with viral and other nonviral transfections with high production costs, cumbersome quality controls, and potential insertional mutagenesis, the nanoparticle gene editing system is an ideal off-the-shelf platform for the fabrication of CAR T cell *in vivo* because of its small size, efficient nucleic acid carrying, customized composition, prolonged blood circulation, and existing large-scale production instruments.^110^ Previous studies have shown that nanoparticles can effectively deliver siRNA, immunomodulatory drugs, and small molecular drugs to CD4^+^ or CD8^+^ T lymphocytes.^111^, ^112^, ^113^ Therefore, affordable and efficient nanoparticle-based gene editing may be a promising strategy to produce CAR T cells *in vivo* (Fig. 4).

### Nanoparticles-based transient transfection

In recent years, mRNA transfection has emerged as a favorable genome transfer technology because of its superior safety, efficient transfection of mitotic and non-mitotic cells, and ease of large-scale production.^114^ However, it faces two challenges, easy enzymatic degradation by ubiquitous RNases and inefficient intracellular delivery.^115^ Fortunately, nanoparticles can ameliorate their instability and improve the transfection rate because of their small size and customized constituents, which typically include cationic and ionizable lipids to encapsulate mRNA into the inner core via electrostatic interactions and assist endosomal escape through protonation.^116^^,^^117^ So far, nanoparticle-based mRNA transfection has shown promising preclinical results in a range of diseases due to its high transfection efficacy, rapid treatment response, and controllable adverse events, especially *in vivo* transfer. Early research has proven the feasibility of delivering mRNA to immune cells *in vivo* to produce passive vaccination against cancer.^118^ Thran and coworkers treated Raji lymphoma with lipid nanoparticles encoding rituximab mRNA, which significantly decreased or even abolished tumor cell growth. In addition, this strategy has achieved success in transferring anti-rabies and antitoxin mRNA *in vivo* to prevent and treat rabies infection and botulinum intoxication, respectively. Published data have also demonstrated the feasibility of *in vivo* delivery of modified mRNA into T cells to produce bispecific antibodies.^119^ Stadler and colleagues constructed mRNA encoding bispecific antibodies that directly targeted CD3 and ovarian carcinoma-associated antigen claudin 6 (CLDN6) and eliminated OV-90 cancer cells upon consecutive injection of a polymer/lipid-based transfection reagent carrying CD3 × CLDN6 mRNA.

Nanoparticle-based mRNA transgenes have also been successfully used to produce CAR T cells for the treatment of various diseases. Epstein et al produced antifibrotic CAR T cells *in vivo* to treat cardiac injury by injecting CD5-targeted lipid nanoparticles carrying modified mRNA.^51^ They produced CD5-targeted lipid nanoparticles encapsulating CAR mRNA against fibroblast activation protein (FAP), and this nanoparticle delivery system exhibited transfection rates of 83% and 17.5%–24.7% *in vitro* and *in vivo*, respectively. Among the FAPCAR T^+^ cells, CD4^+^ and CD8^+^ T cells accounted for 87% and 9%–10%, respectively. Importantly, most CD4^+^ and CD8^+^ T cells present a naïve phenotype, which is beneficial for CAR efficacy.^120^ Lastly, they demonstrated potent anti-fibrosis activity and cardiac function restoration after treatment with the modified nanoparticles in a mouse model of heart failure. The transient expression of non-tumor-targeted CAR not only cured the disease but also avoided latent adverse events, which provided significant promise for various other diseases. Similarly, Parayath et al reported transient reprogramming of circulating T cells *in vivo* upon injection of nanoparticles carrying tumor-specific CAR or T cell receptor (TCR) mRNA.^52^ They synthesized a biodegradable poly(β-amino ester) (PBAE) polymer formulation packaging *in vitro*-transcribed mRNA and then functionalized PBAE with T-cell-targeted (CD8 or CD3) antibodies to successfully produce CAR T or TCR-T cells *in vivo*, which showed similar anti-cancer responses to human leukemia, prostate cancer, and hepatitis B-induced hepatocellular carcinoma as *in vitro* engineered T cells. Importantly, this off-the-shelf CAR T product is as convenient as conventional drugs, allowing more patients to benefit from this immunotherapy. However, the maximum levels of CAR or TCR expression occurred on day 2 after transfection and rapidly decreased thereafter, maintaining expression for approximately 7 days. Additionally, Zhao and colleagues applied virus-mimetic fusogenic nanovesicles carrying CAR protein to produce CAR T cells via membrane fusion *in vivo*.^121^ These techniques were transient expression of CAR, and repeated administration was necessary to achieve efficient anti-tumor activity.

### Nanoparticles-based persistent transfection

In addition to transient transfection, persistent transfection is another critical method to produce CAR T cells *in vivo*, and it can avoid repeated administration. Smith and colleagues first reported *in situ* reprogramming of circulating T cells to persistently produce CAR T cells based on synthetic DNA nanocarriers.^41^ They synthesized PBAE polymer packaging DNA encoding CD19 CAR and functionalized it with anti-mouse CD3ε F(ab')2 fragments and microtubule-associated nuclear localization sequences to target T cells and the nucleus, respectively. In addition, this nanoparticle system contained a plasmid encoding transposase iPB7 to integrate CAR DNA into chromosomes. *In vitro* results suggested that CD19 CAR could be detected on T cells at 30 h post-transfection, and the mean transfection rate was 3.8%; *in vivo* experiments indicated similar anti-leukemia activity compared with conventional LV transfection *in vitro*. Notably, the subtypes of nanoparticle-internalized T cells were mostly naïve phenotypes with superior anti-tumor activity.^122^ This first report unveiled the prelude to producing CAR T cells *in vivo* and boosted its competitiveness with frontline pharmaceuticals, such as small-molecule targeted drugs,^123^ owing to increased stability and affordable costs. In a study conducted by Yu et al, plasmid DNA-loaded self-assembled nanoparticles were fabricated based on adamantane-grafted polyamidoamine dendrimers and cyclodextrin-grafted branched polyethyleneimine.^124^ They successfully delivered the epidermal growth factor receptor variant III-CAR into Jurkat T cells and showed specific anti-tumor activity.

### Limitations of nanoparticles-based transfection

Despite the successes achieved in nanomedicine to produce CAR T cells *in vivo*, the following limitations might hamper their broad application in clinical practice. Successful transient gene editing *in vivo* must target cell uptake, endosomal escape, and nucleic acid release, while persistent expression must enter the nucleus in addition to the above conditions. On the one hand, gene delivery efficacy is significantly lower than that of viral vectors due to the above cellular barriers,^59^ especially for persistent transfection. On the other hand, a low transfection rate leads to unavoidable repeated administration to produce sufficient CAR T cells to kill cancer cells. In addition, nanoparticles carrying CAR typically consist of multiple ingredients, and minor variations may influence their physicochemical characteristics and intended functions. Therefore, large-scale production of nanoparticles should be performed under good manufacturing conditions and strict fabrication rules to obtain safe, stable, and reliable nanomedicines.

## Conclusions and perspectives

Recently, the *in vivo* CAR T cell therapy has attracted continuous interest owing to its simple manufacturing process, controllable batch-to-batch variability, and “off-the-shelf” characteristics. More importantly, *in vivo* CAR T cells can be produced on a large scale, which may reduce costs and broaden clinical applications. Despite the remarkable advantages of *in vivo* CAR T cell therapy, the safety profile, transfection rates, and clinical efficacy should be further improved for application in sizable populations. At present, all CAR T cell therapies approved by the U.S. FDA are produced by viral vectors, which carry potential tumorigenic risk due to random integration of CAR. Therefore, achieving the targeted integration of CAR *in vivo* may be the future research direction. To improve the efficacy and safety of *in vivo* CAR T cell therapy, the following strategies may be feasible.

Targeted integration or non-integrating DNA — Zhang et al developed a novel type of CAR T cell by inserting CAR sequence into the PD-1 locus of T cells, exhibiting superior anti-tumor ability in patients with B cell non-Hodgkin lymphoma.^125^ To avoid genomic integration of external plasmids, Bozza et al reported an interesting study in which scaffold/matrix attachment regions (S/MARs), DNA sequences dividing the chromatin into structural and functional domains,^126^ were used to mediate extrachromosomal replication of non-integrating DNA vectors in dividing cells.^127^ This platform included no immunogenic components and ensured persistent gene expression in human T cells without impairing their molecular integrity and activity. In addition, they developed a manufacturing protocol to rapidly produce approximately 3.3 × 10^8^ CAR T cells from 1 × 10^9^ purified T cells in 5 days and showed enhanced anti-cancer activity compared with conventional lentivirus vectors. Notably, they used a transfection system containing no immunogenic composition, which was significantly safer than the current viral vector. Therefore, S/MARs-mediated nonviral and non-integrating DNA delivery is a promising method to produce safer CAR T cells *in vivo*.

Combination — a variety of technologies could be combined to benefit from each other's strengths to achieve a higher transfection rate and clinical efficiency. Raes et al demonstrated that vapor nanobubble photoporation is an auspicious physical transfection method that combines gold nanoparticles with a pulsed laser, resulting in an effective mRNA transfection rate of up to 45%.^128^ Similarly, Xu and colleagues combined photothermal therapy and CAR NK therapy to thoroughly eliminate lung cancer.^129^ In addition, Sterner and colleagues applied CRISPR/Cas9 to disrupt the granulocyte-macrophage colony-stimulating factor of CAR T cells, resulting in the abrogation of neuroinflammation and enhanced anti-cancer activity.^130^ Interestingly, Qu and coworkers found that multiple myeloma cell membrane-encapsulated nanoparticles not only targeted cancer cells through homologous targeting but also escaped phagocytosis of the mononuclear phagocyte system to prolong the circulation time.^131^ Packaging nanoparticles with the cell membrane may be a promising platform to increase the transfection rate and reduce off-target effects. Given the synergistic effects mentioned above, it is hoped that clinical efficiency can be boosted by combining *in vivo* CAR T cells with other therapeutic strategies.

Other CAR immune cells — The confined infiltrating capacity and inhibitory effect of the tumor microenvironment result in limited effects of CAR T cells on solid tumors. However, other CAR immune cells, such as CAR macrophages and CAR NK cells, have shown promising results in the treatment of solid tumors. Klichinsky et al proved that CAR macrophages could eliminate cancer cells in a human ovarian cancer xenograft mouse model and convert M2 macrophages to M1 macrophages by secreting proinflammatory cytokines and chemokines.^132^ In addition, CAR NK cells were able to overcome the tumor microenvironment and kill solid tumors, and cord blood-derived NK cells could enhance the anti-tumor activity of CAR T cells.^133^^,^^134^ Importantly, nanoparticle-mediated *in vivo* production of CAR M1 macrophages significantly suppressed tumor growth in a neuroblastoma mouse model and could present tumor antigens to naïve T-cells.^135^

In conclusion, the above methods are promising methods to address the current challenges of *in vivo* CAR T cell therapy and produce safer, more stable, and more efficient CAR T cells to make more patients benefit from CAR T cell therapy.

## CRediT authorship contribution statement

**Zhiqiang Song:** Writing – original draft, Visualization, Investigation. **Yi Zhou:** Writing – original draft, Visualization, Investigation. **Binbin Wang:** Writing – original draft, Visualization, Investigation. **Yuke Geng:** Visualization, Investigation. **Gusheng Tang:** Writing – review & editing, Supervision, Conceptualization. **Yang Wang:** Writing – review & editing, Supervision, Conceptualization. **Jianmin Yang:** Writing – review & editing, Supervision, Conceptualization.

## Funding

This work was supported by Science and Technology Commission of Shanghai Municipality (China) (No. 24YF2758000), National Natural Science Foundation of China (No. 82270202, 82300257, 82470190), Medical-enterprise Integration Innovation and Collaboration Project (China) (No. SHDC2023CRT005), Youth Start-up Foundation of the First Affiliated Hospital of Second Military Medical University (China) (No. 2022QN067), Changhai Hospital "Changfeng" Project (China), and Changzheng Hospital "Pyramid Talent" Project (China).

## Conflict of interests

All authors declared no competing interests.
