# Supplementary material for: In vivo production of CAR T cell: Opportunities and challenges
Source: Genes Dis. 2025 Mar 25;12(6):101612. doi: 10.1016/j.gendis.2025.101612 (PMC12357247; doi:10.1016/j.gendis.2025.101612)
Supplement: Multimedia component 1 [file mmc1.docx]

**Table S1.** Summary of major advantages and challenges of various *in vivo* CAR T cell production techniques.

| **Delivery vector** | **Advantage** | **Challenge** |
| --- | --- | --- |
| AAV | Low risk of insertional mutation and multiple-target subcategories | Small packaging capacity |
| LV | Large packaging capacity and long-term transgene expression | Insertional mutation |
| Electroporation | High transfection rate even in difficult transfect cells | Elusory electroporation parameters and tissue damage for human |
| Nanoparticles | Low immunogenicity | Toxicity and low transfection rate |
| Engineered proteins | Small size and easy production | Immunogenicity |

AAV, adeno-associated virus; LV, lentiviral vectors.
